# A High-Computational Efficiency Human Detection and Flow Estimation Method Based on TOF Measurements

**DOI:** 10.3390/s19030729

**Published:** 2019-02-11

**Authors:** Weihang Wang, Peilin Liu, Rendong Ying, Jun Wang, Jiuchao Qian, Jialu Jia, Jiefeng Gao

**Affiliations:** Brain-inspired Application Technology Center, School of Electronic Information and Electrical Engineering, Shanghai Jiao Tong University, Shanghai 200240, China; weihangwang@sjtu.edu.cn (W.W.); rdying@sjtu.edu.cn (R.Y.); wj65832869@sjtu.edu.cn (J.W.); jcqian@sjtu.edu.cn (J.Q.); jjljjl@sjtu.edu.cn (J.J.); gaojiefeng_aarony@sjtu.edu.cn (J.G.)

**Keywords:** TOF, human detection, flow estimation, computational efficiency

## Abstract

State-of-the-art human detection methods focus on deep network architectures to achieve higher recognition performance, at the expense of huge computation. However, computational efficiency and real-time performance are also important evaluation indicators. This paper presents a fast real-time human detection and flow estimation method using depth images captured by a top-view TOF camera. The proposed algorithm mainly consists of head detection based on local pooling and searching, classification refinement based on human morphological features, and tracking assignment filter based on dynamic multi-dimensional feature. A depth image dataset record with more than 10k entries and departure events with detailed human location annotations is established. Taking full advantage of the distance information implied in the depth image, we achieve high-accuracy human detection and people counting with accuracy of 97.73% and significantly reduce the running time. Experiments demonstrate that our algorithm can run at 23.10 ms per frame on a CPU platform. In addition, the proposed robust approach is effective in complex situations such as fast walking, occlusion, crowded scenes, etc.

## 1. Introduction

Accurate human detection and flow estimation attracts a lot of attention because of its vital application in the field of urban public transportation, intelligent building, etc. Such technology enables a new interaction between people. Specifically, flow estimation in public places such as airports, railway stations, and shopping malls can help staff analyze passenger density and channel dense crowds. In addition, the number of people getting on and off buses, metro systems and trains can contribute to the passenger flow analysis. Despite the significance of human detection and flow estimation, it remains a subject of active and challenging research.

Human detection derives from a combination of Histogram of Oriented Gradient (HOG) features and Support Vector Machine (SVM) classification, as shown in [1]. Specific images are exhaustively searched for by a sliding window filter, which generates several candidate regions, resulting in high computational complexity. Thus, research efforts attempt to speed up the human detection process. Zhu et al. [2] combine the rejection cascade approach to reduce computation. Beleznai C. et al. [3] propose computationally efficient detection based on shape templates using contour integration by means of integral images, which are built by oriented string scans. Leo M et al. [4] achieve foreground people tracking using a Hidden Markov Model based on blob geometrical information. Demirkus et al. [5] consider geometric modelling in relation to pedestrian detection in fish-eye top-views for the first time. Selective search [6] uses an underlying image structure to generate a set of region hypotheses. The hypothetic regions are merged in terms of similarity of features in color, texture, size, and shape compatibility. Despite the obvious decrease of candidate windows, selective search is still time consuming. With the rise of neural networks, recent research executed on the GPU platform have further reduced the computational complexity [7,8,9]; it, however, continues to be a bottleneck for methods based on deep neural networks using RGB images.

In addition to computational efficiency, multi-scale, overlap, and occlusion are also challenges faced by RGB image-based methods. Moreover, the recognition results of RGB images are greatly affected by external environment changes, such as scene perspective distortions, illumination changes, and color variations. To address the aforementioned issues, a Time-of-Flight (TOF) camera becomes another choice. 

TOF cameras produce depth images by measuring the phase difference between the radiated and reflected IR waves directed to the target object, each pixel of which encodes the distance to the corresponding point in the scene. Compared to RGB images, depth images measured by a TOF camera exhibit several advantages for human detection. In spite of low resolution, depth images provide more geometrical information. Features extracted from depth images are commonly not affected by scale, rotation, and illumination [10]. An estimated 3D pose of an object from depth images is more accurate, compared to an estimated pose from RGB images [11]. Moreover, TOF camera not only preserves privacy, but also achieves object segmentation in a more straightforward way than the traditional RGB cameras. Accordingly, depth images have the potential to overcome many difficulties faced by RGB images on human detection [12]. In addition, TOF is highly adaptable to harsh lighting conditions and external environment changes. Conventional TOF cameras rely on a front-facing setup that suffers from occlusion if multiple people interact with each other simultaneously [13]. The top-view TOF camera can solve the problem of multi-scale and occlusion under the front-view images. 

Recent human detection approaches based on top-view TOF measurements can be divided into two broad categories, namely geometric model based and feature based. For the model-based approaches, Zhang et al. [14] assume that the head is the closest body part to the vertical TOF camera and proposed head detection based on the local minimums of the depth image. This method is scale invariant, but cannot handle situations such as raising a hand over the head. Template matching is applied to locate head and shoulder patterns and split the fused blobs as individual people [15]. However, in the case of crowding people, this method is limited and cannot accurately separate the detected hull into multiple persons. Similarly, the method [16] projects the point cloud onto the ground plane to find people blobs. It also faces the problem that the projection may result in fused blobs for close people in extremely crowded scenes. A dynamic Gaussian Mixture Model (GMM) is used to extract the moving foreground objects as people in [13]. In addition, graph-based segmentation [17] separates the top-view human shape into a head region and other connected regions. As for complex scenes with high image noise, segmentation based on GMM and graph are not feasible. The other category is feature-based human detection. These methods, rather than relying on an explicit detection process to describe and identify persons in depth image, extract features to classify the candidate regions into people and other objects. A hemi-ellipsoid model is introduced to especially describe the 3D appearance of the upper head in [17]. A 5-dimensional vector, composed of the fitted hemi-ellipsoid parameters, describes the configuration of head shapes. However, objects, such as floor lamps and basketballs, whose shapes are similar to the head surface model probably lead to false detection. Another approach proposes local maxima search and mean-shift clustering to generate sufficient head candidates. Moreover, a depth feature descriptor, based on SLTP feature [18], computes relative depth differences as the input for SVM in [19]. This method can respond to many challenging situations with humans tailgating and piggybacking. However, it generates excessive human candidates, resulting in high false positives and computational costs. Moreover, the proposed feature is insufficient to precisely represent the characteristics of the 3D head region shapes. Therefore, methods like [17,18,19] need further processes to distinguish between human and other objects. A set of criteria on depth values and orientations in adjacent neighborhood areas is designed in [20], in order to estimate the individual region of interest (ROI). Besides, a six-dimensional histogram feature, composed of the number of pixels in the head, neck and shoulder area, discriminates between people and other objects. Nevertheless, the components of the feature vector are sensitive to the appearance changes of people, such as hairstyle, hair length, and so on. Hence, the common problem of [17,18,19,20] is how to extract features that really describe the characteristics of the head region. 

For the tracking algorithm, a height-based matching algorithm is proposed in [21]. Bondi et al. [17] use a greedy approach that iteratively associates track/detection pairs with the smallest distance. A constant speed model is used to perform the probabilistic filtering and tracking based on extended particle filter with a clustering process in [20]. The kalman-based multi-object tracking of coordinates and velocity of each detected object is proposed to match and update the tracks [22]. Besides, a weighted K Nearest Neighbor based multi-target tracking method is adopted to track each confirmed head and count people through the surveillance region [23]. The corresponding weights are calculated by the Gaussian function of distance between head position point and K nearest trajectories. These matching algorithms based on single features cannot cope with complex scenes such as occlusion.

In this paper, we present a real-time framework for human detection and flow estimation using top-view TOF camera measurements. The proposed algorithm mainly consists of three modules: head detection based on local pooling and searching, classification refinement based on human morphological features, and tracking assignment filter based on dynamic multi-dimensional features. Local pooling dramatically reduces the amount of computation, while preserving local key information for the depth image. In addition, the multi-dimensional feature combines the spatial relationship and height of human candidate points by a penalty function. The common constraint of multiple features make the trajectory assignment and update more accurate. As the previous datasets are all captured by front- or side-view camera, we contribute a new dataset from top-view camera for human detection and flow estimation. The new dataset includes more than 10k entries and departure events with detailed human location annotations. The experiments demonstrate that our method achieves high accuracy (97.73%) and reduces the running time significantly with 23.10 ms per frame on a CPU platform. In addition, the proposed robust approach is still effective in complex situations such as fast walking, occlusion, crowded scenes, etc.

This paper is organized as follows. Section 1 provides a general introduction and review of the related literature. Section 2 includes the detailed human detection and flow estimation approach. The experimental setup and results are introduced in Section 3. Finally, Section 4 states the conclusions and future work.

## 2. Human Detection

Human detection consists of three main modules: preprocessing, local pooling and searching, and classification refinement. First, the preprocessing step fills the invalid pixels and smoothens the depth image. Then, human position candidates are hypothesized by local pooling and searching. Finally, head diameter estimation based on height and head shape classification, based on human morphological features, refine the head candidate regions. The whole module generates sufficient and accurate human candidate proposals, as few as possible. In this section, each stage of the people detection method is described in detail as follows. 

### 2.1. Preprocessing

**Figure 1:**
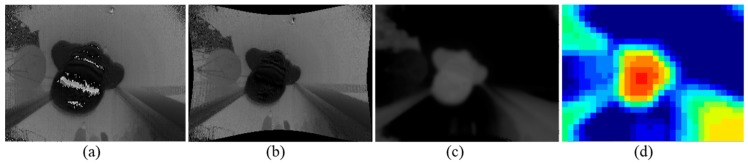
Distortion rectification and Gaussian filtering. (**a**) Raw depth image with distortion and overexposure; (**b**) corrected and rectified depth image. The objects in the scene are adjusted to the correct size. The overexposed pixel values have been corrected; (**c**) depth image with pixel values converted to the ground plane; (**d**) Visualization of pooled image. The red area symbolizes proximity to the TOF camera. Blue pixels mean far distance from the TOF camera.

The TOF camera is mounted on a top-view location, with the optical axis vertical to the floor plane. Before the vision algorithm, we need to deal with the noise of the TOF measurements. Figure 1a presents the raw depth image measured by overhead TOF camera with noise. There are two main causes for noise value of depth images. One is overexposure due to the excessive integration time. The other is the light absorption fact of black objects, making the reflection light too weak to acquire a correct distance. The distorted pixels of the depth image are indicated as white points shown in Figure 1a.

To reduce noise and invalid value pixels, we propose a padding and filtering method to improve the quality of the depth images. Due to the principle of lens imaging, image distortion is widespread in all kinds of cameras. Distortion changes size and shape of the objects in the depth image. Hence, rectification is a necessary operation for depth image processing. Equation (1) shows the relationship between real location (x, y) and distortion location $\left(x^{\prime },\;y^{\prime }\right)$.

(1) $$\left[\begin{array}{c} x^{\prime }\\ y^{\prime } \end{array}\right]=\left(1+k_{1}r^{2}+k_{2}r^{4}+k_{3}r^{6}\right)\left[\begin{array}{c} x\\ y \end{array}\right]+\left[\begin{array}{c} 2p_{1}xy+p_{2}\left(r^{2}+2x^{2}\right)\\ 2p_{1}\left(r^{2}+2y^{2}\right)+2p_{2}xy \end{array}\right]$$

where $\left[k_{1},\;k_{2},\;k_{3},\;p_{1},\;p_{2}\right]$ is distortion coefficients matrix. 

Remapping the distortion location $\left(x^{\prime },\;y^{\prime }\right)$ into the real location (x, y), we can get corrected and rectified pixel (u, v) by coordinate transformation (2).

(2) $$\left[\begin{array}{c} u\\ v\\ 1 \end{array}\right]=\left[\begin{array}{ccc} f_{x} & 0 & c_{x}\\ 0 & f_{y} & c_{y}\\ 0 & 0 & 1 \end{array}\right]\cdot \left[\begin{array}{c} x\\ y\\ 1 \end{array}\right]$$

where $\left[\begin{array}{ccc} f_{x} & 0 & c_{x}\\ 0 & f_{y} & c_{y}\\ 0 & 0 & 1 \end{array}\right]$ is the internal matrix of the TOF camera. 

In this work, we measured the distortion coefficients matrix and camera matrix of TOF. On this basis, we fill the invalid pixels in the depth images. For each depth image, traversal on the entire image finds the invalid pixels and sets the value as zero. Figure 1b shows the corrected and rectified depth images. Then, a Gaussian filter with kernel size of 9×9 smoothens the depth image. The invalid pixel values are rectified by the nearest neighborhood area. A maximum depth value filter is designed for each invalid measurement pixels. The filter takes the maximum value in the 3×3 neighborhood in place of the current pixel value, for each invalid pixel point. The series of operations ensures that invalid pixels are accurately corrected in the depth images. 

Image correction results in the black boundary around the depth image. In order to eliminate these black areas, we made an 8-bit-scale mask to distinguish the image area and boundaries. Through the division operation of the depth image and the mask, the black border around the image is approximately substituted by the corresponding area in the original depth image. Gaussian filtering of the 8-bit-scale mask ensures that the denominator of the division is not zero.

(3) $$mask\left(i,j\right)=\{\begin{array}{c} 255,\left(i,j\right)\in image\;area\\ 0,\left(i,j\right)\in boundary\;area \end{array}$$

In order to adapt to different installation heights, we convert the image depth values from the distance to the TOF camera into the height to the ground. According to the deployment height $h_{D}$, the value of each pixel (i,j) is calculated as Equation (4). Figure 1c exhibits the results of depth image conversion.

(4) $$d_{\left(i,j\right)}=h_{D}-r_{\left(i,j\right)}$$

where $d_{\left(i,j\right)}$ is the pixel value converted to the ground plane. $r_{\left(i,j\right)}$ is the pixel value of previous corrected depth image.

### 2.2. Local Pooling and Searching

For the top-view perspective of a human candidate, there is no doubt that the head is one of the most significant features in human detection. Hence, human location candidates are proposed according to head features in the depth images. In this module, sufficient head position candidates are generated by local pooling and searching.

**Figure 2:**
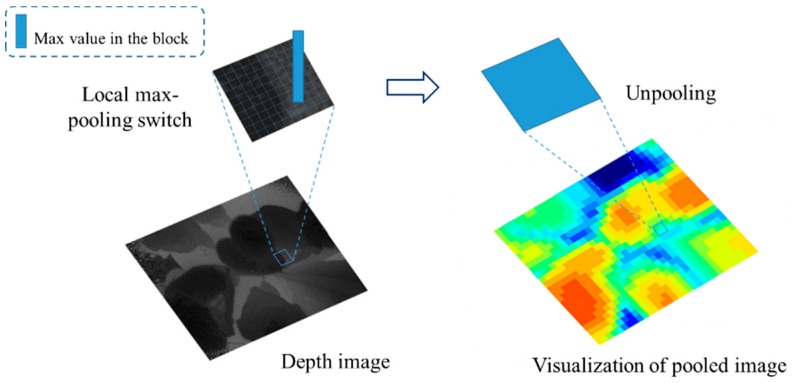
Local pooling. For visualization, we resize the pooled image to 320 × 240. Each pixel in the pooled image is a 10 × 10 block.

In order to improve computational efficiency and only use the most valuable information, the depth image is down-sampled into a smaller size. The process is named local pooling and the image obtained by local pooling is named pooled image. The local pooling divides the depth image into 32×24 blocks in size of 10×10. Similar to max-pooling in neural network, local pooling only reserves the pixel with maximum value in the current block. Therefore, the resolution of the pooled image reduced from the original 320×240 to 32×24. At the same time, the pixel value of each point of the pooled image is reduced to one hundredth of the original value as an integer. Figure 2 shows the result of pooled images.

In the top-view scene, the human is supposed to be the local maximum region in the neighborhood. Therefore, we find local maxima points in the 5×5 neighborhood on the pooled image. Since pixel values are normalized in the pooled images, more than one local maximum points probably exist in the neighborhood. The result of the local maximum is supposed to be several connected regions. However, some local maxima regions with low height less than the threshold are discarded, eliminating other non-human objects on the ground. Then, the connected regions are separated individually and labeled with different index. According to the 8-connectivity rule, forward scan mask is used to avoid showing repeated comparisons. That is, connectivity detection is executed by checking the labels of neighbor pixels among the north-east, north, north-west, and west of the current pixel. In this work, we use the fast optimizing connected component labeling method proposed by Wu et al. in [24]. The algorithm performs two passes over the image. The first scan over the image assigns provisional labels and establishes equivalence information. Then, passing through the image again replaces each temporary label by the smallest label of its equivalence class and assigns the final label. Next, the center point of each connected region is calculated as the initial head position candidate.

**Figure 3:**
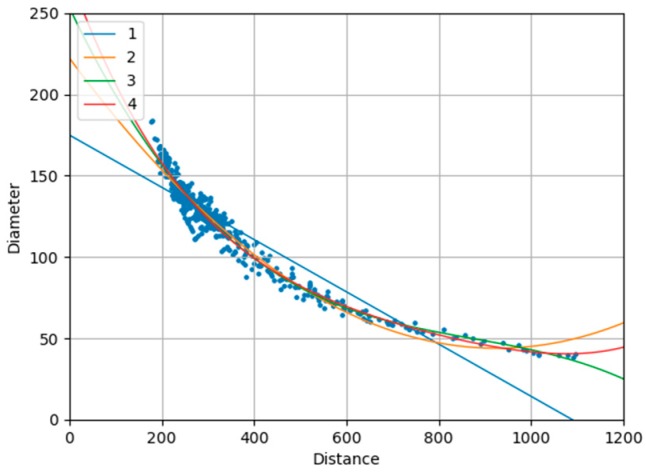
Polynomial equation fitting with different degrees.

**Figure 4:**
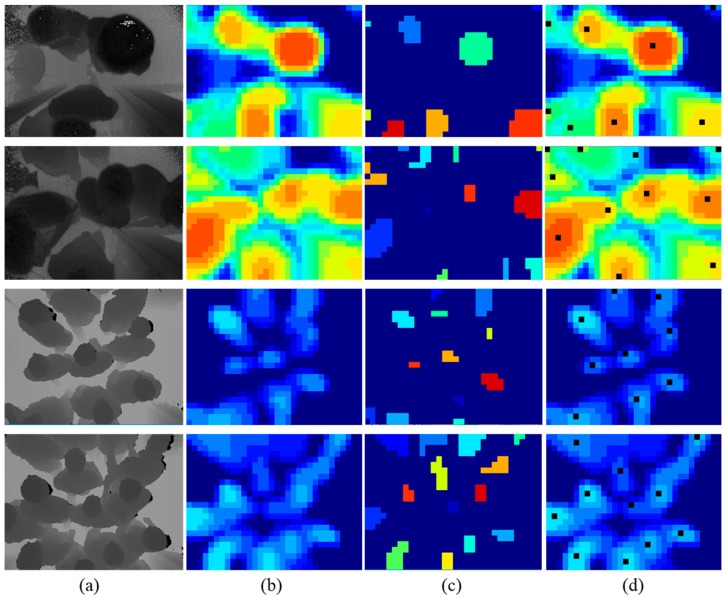
Local pooling and searching. (**a**) The input depth images from simple to complex scenes; (**b**) visualization of pooled images; (**c**) visualization of local searching and connect component labelling; (**d**) results of the human detection module.

The geometric center of the concave polygon may be outside the shape and may fall in the background. Therefore, the coordinate mean of each connected region is computed as the cluster center. In addition, the relationship between head diameter and distance to the TOF camera is fitted by more than 4000 samples on people of different heights, gender, and wear. This relationship is highly robust because it does not change with the variation of the installation height of TOF camera. For each head region, the head center position and the two endpoints of head diameter line are manually labelled. We use pixel value at head center to calculate the distance from the person to the TOF camera, and calculate the distance between two endpoints as the true value of the head diameter. We evaluate polynomial equation model with different orders and finally select the 3-order polynomial with the minimal Root Mean Square Error (RMSE), regarded as the best fitting performance. The fitting polynomial equation is presented in (5). Figure 3 shows the fitting effects based on various degree parameters. Head diameter fitting helps determine the area of the head region. In each head region, only the candidate point with the highest height is retained. Finally, all the remaining candidates serve as the head positions results. The results of each step in local pooling and searching are shown in Figure 4.

(5) $$y=233.1752-0.5968x+6.2522\times 10^{-4}x^{2}-2.3854\times 10^{-7}x^{3}$$

where y is the estimated diameter of head. x is the distance to the TOF camera.

### 2.3. Classification Refinement

Certain non-head objects (false positive head positions) have similar features to real people in depth images. The non-head objects are likely to be mistakenly detected on the basis of depth information. Therefore, we design a shallow CNN to remove non-head candidate points through classification.

**Figure 5:**
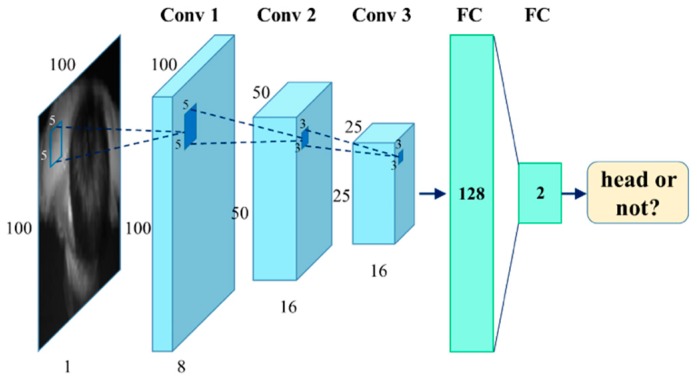
Architecture of the classification refinement network.

The input of the shallow CNN is a single-channel depth image block with resolution of 100×100, whose center is the proposed head position point. Figure 5 illustrates the architecture of the network. The model contains three convolution layers and two fully connected layers. Conv1 has 8 5×5 convolutional filters with stride 2. Both conv2 and conv3 have 16 3×3 convolutional filters with stride 2. All convolutional layers are followed by a 2×2 max pooling layers for down-sampling. Rectified linear unit (ReLU) is the activation function. The feature map of conv3 layer is mapped to a low-dimensional feature. This feature is fed into the classification layer with softmax function. In addition, dropout layer is added after the FC layer, which randomly disconnects a fraction rate of input units to speed-up the forward propagation in the prediction process. Meanwhile, the dropout layer efficiently prevents overfitting during the training process.

Instead of end-to-end deep neural networks, our method reduces the computational cost significantly with less trainable parameters. Compared with U-Net3 [25], which has 1.9 million trainable parameters in total, and ResNet [26], owning more than 2.7 million trainable parameters, the number of trainable parameters of our classification network is only 4914. Thus, our method effectively reduces the computational burden and achieves real-time performance. It is worth noting that in order to ensure a sufficiently high recall rate for the tracking module, the probability decision threshold of the classification refinement is not set too high. In conclusion, the classification refinement strategy makes our algorithm perform well in crowd scenes such as when people are close to each other, wave hands, hold a newspaper, and other partial occlusion scenarios.

## 3. Flow Estimation

As mentioned in the introduction, most research only focuses on the human detection task. In this work, we introduce a novel track assignment filter across frames, based on multi-dimensional feature matching. Inspired by the scan-line algorithm [27], an effective matching strategy is designed to accelerate the flow estimation module, which is much faster than greedy search [28] and KNN [23]. Finally, the number of people entering and leaving the scene are counted accurately.

### 3.1. Track Assignment Filter

In this work, the head locations proposed by the human detection module are the inputs of track assignment filter in the current frame. Some (or all or none) head position points are selected by the filter across frames to join the existing trajectory. The other head position points are judged on whether they satisfy the condition of starting point of a new trajectory, which will be introduced in detail in the following subsections. The tracks update in each frame for further matching. 

Given positions $P^{m}$ of m heads in the current frame and n existing trajectories $T^{n}$, we match each head position point with the last points $L^{n}$ of all the *n* trajectories.

(6) $$P^{m}={\left[\left(x_{1}^{p},y_{1}^{p}\right),\left(x_{2}^{p},y_{2}^{p}\right),\ldots ,\left(x_{m}^{p},y_{m}^{p}\right)\right]}^{T}\;L^{n}={\left[\left(x_{1}^{l},y_{1}^{l}\right),\left(x_{2}^{l},y_{2}^{l}\right),\ldots ,\left(x_{n}^{l},y_{n}^{l}\right)\right]}^{T}$$

We define a covariance matrix C of *m* current head position points and *n* last points of existing trajectories with height. The value of each element $c_{ij}$ is the difference between two points $\left(x_{i}^{p},y_{i}^{p}\right)$ and $\left(x_{j}^{l},y_{j}^{l}\right)$, calculated by combining Euclidean distance and height difference features.

(7) $$C={\left(c_{ij}\right)}_{m\times n}=\left[\begin{array}{ccc} c_{11} & c_{12} & \begin{array}{cc} \ldots & c_{1n} \end{array}\\ c_{21} & c_{22} & \begin{array}{cc} \ldots & c_{2n} \end{array}\\ \begin{array}{c} \vdots \\ c_{m1} \end{array} & \begin{array}{c} \vdots \\ c_{m2} \end{array} & \begin{array}{cc} \ddots & \vdots \\ \ldots & c_{mn} \end{array} \end{array}\right]$$

(8) $$c_{ij}=d\left(p_{i}^{m},l_{j}^{n}\right)={\left\|p_{i}^{m}-l_{j}^{n}\right\|}_{2}+\lambda \phi \left(h_{i}^{p},h_{j}^{l}\right)\;{\left\|p_{i}^{m}-l_{j}^{n}\right\|}_{2}=\sqrt{{\left(x_{i}^{p}-x_{j}^{l}\right)}^{2}+{\left(y_{i}^{p}-y_{j}^{l}\right)}^{2}}\;\phi \left(h_{i}^{p},h_{j}^{l}\right)=\max\left(0,\;⎣|h_{i}^{p}-h_{j}^{l}|-\varepsilon ⎦\right)$$

where $h_{i}^{p}$ is the height of $i^{th}$ head position point in the current frame. $h_{j}^{l}$ is the height of last point of $j^{th}$ existing tracks. $p_{i}^{m}$ denotes $\left(x_{i}^{p},y_{i}^{p}\right)$ and $l_{j}^{n}$ denotes $\left(x_{j}^{l},y_{j}^{l}\right)$.

The height difference is used as the penalty term to ensure height consistency of a trajectory. The matching is permitted only if the height difference between the head position point and the end point of existing tracks is less than the threshold ε. In addition, λ denotes the penalty parameter, set as 1000, and ε is set as 2 empirically.

**Figure 6:**
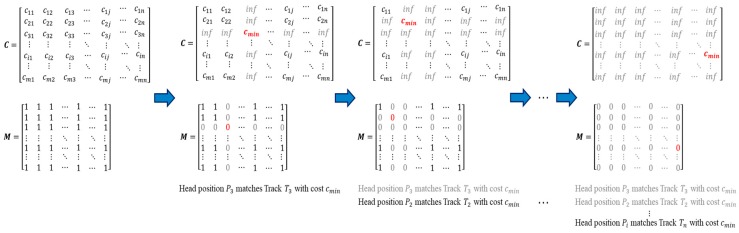
The procedure of computing elements in the covariance matrix ***C*** and mask ***M***.

After obtaining the covariance matrix C, we search the associated head position point and trajectory pairs with minimum distance. Unlike the conventional multiple global exhaustive search, we first define a binary mask M with the same shape of C and find the matching pair with minimum distance. The elements in M are initialized as one. Once the $i^{th}$ head position point matches the $j^{th}$ trajectory, the $i^{th}$ row and the $j^{th}$ column of the mask M are set to 0, and the corresponding value of the covariance matrix C is set to infinity. In other words, these elements are considered as invalid areas and no longer involved in the subsequent processing. For the next iterations, only the valid areas are processed until all the elements in the mask M change to 0. The procedure of computing the element in the covariance matrix C and mask M is shown in Figure 6. Finally, a filter result matrix $D_{m\times 2}$ with matching distance and index of corresponding matching trajectory is obtained. For each head position point, if the matching distance is less than the pre-defined threshold, the current point is merged into its corresponding trajectory. The remaining unmatched head position points are checked to see if they identify as the starting point of the new track by the counting strategy. Besides, the unassociated trajectories are temporarily disabled. The counting module will determine if the current track is in an ending state. The track assignment filter selects several true head positions to join the existing tracks. The filtering process is presented in the form of pseudocode below.

**Algorithm 1.** Track Assignment Filter**Input:** Covariance Matrix $C_{m\times n}$, Head Position $P^{m}$
**Output:** Matching Distance and Index Matrix $D_{m\times 2}$, Mask Matrix $M_{m\times n}$
1: **initialize:** Set $M_{m\times n}=1_{m\times n}$, $D_{m\times 2}=\infty_{m\times n}$
2: 
**while**
$M_{m\times n}\neq O_{m\times n}$
**do**
3:   Compute the matching pair index ***i***,***j*** with minimum distance cost θ;4:   $M_{*j}=0$ and $M_{j*}=0$;5:   $d_{i1}=\theta$ and $d_{i2}=j$;6:   $c_{*j}=\infty$ and $c_{i*}=\infty$;7: 
**end while**
8: **for** each head position $p_{i}^{m}\in P^{m}$
**do**9:   **if** Distance Cost $d_{i1}<\tau$
**then**10:    $p_{i}^{m}$ joins the corresponding Track $d_{i2}$;11:   **else**12:    Determine whether being new track or not13:   **end if**14: 
**end for**
15: **return**$D_{m\times 2}$ and $M_{m\times n}$


### 3.2. Counting Strategy

In order to make the counting strategy more universal in various scenes, we define two lines as the boundary line for incoming and outgoing events. If someone crosses the two lines, it proves that the person has entered or left the scene. As the resolution of the depth image is 320×240, the two boundaries are set to y=80 and y=160*,* respectively. Three criteria are proposed for the starting point of a new trajectory. Firstly, the point $\left(x_{s},y_{s}\right)$ does not match any existing tracks. Besides, the distance between the point and any existing trajectory is more than the pre-defined threshold ξ. The parameter ξ is set as 5 empirically. This operation can prevent accidental trajectory interruption, in the case of mistakenly detecting the head and back as two head positions. Moreover, the point is supposed to be outside the detection line, which meets Equation (9). If three conditions are satisfied simultaneously, this head position point can serve as the starting point of a new candidate trajectory. If the proposed candidate track matches three head position points in succession, the existence of this trajectory is confirmed and updated continually. Then, the tracking and counting strategy starts.

(9) $$y_{s}\in \left(0,\;80\right)\cup \left(160,\;240\right)$$

**Figure 7:**
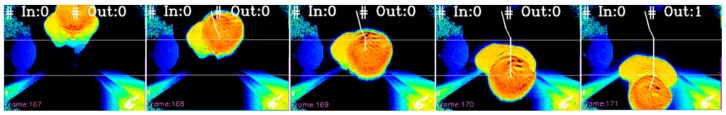
Schematic representation of the counting strategy.

When the track assignment filter leaves unassociated trajectories, tracking state detection is used to determine whether the track ends or not. If no new head position point matches the current trajectory in three consecutive frames, the track no longer updates. Two criteria are defined for detecting the events of entering and leaving the scene. One is the consistency of movement direction. The trajectory needs to move in the same direction in general. The other is the principle of crossing the detection boundaries. The ending point $\left(x_{e},y_{e}\right)$ of a track is supposed to meet the condition in Equation (10). If the two conditions are satisfied simultaneously, this trajectory can be used for flow estimation. Each effective trajectory represents the entry or departure of a person. The direction of entering and leaving is determined based on the coordinate difference between the start and end positions. Figure 7 shows the counting process for the people flow estimation.

(10) $$y_{e}\in \left(0,\;80\right)\cup \left(160,\;240\right)$$

## 4. Experiment

In this section, we define several sets of comparative experiments to evaluate the efficiency and accuracy of the proposed approach. Specifically, the construction of the proposed dataset is introduced, including the experimental environment and parameters of the TOF camera. In addition, the performance of our approach on human detection and flow estimation is evaluated on several datasets, with comparison to the state-of-the-art methods. Our method is implemented in python and run on an Intel Core i5 CPU platform with 8G memory. 

### 4.1. Dataset

**Figure 8:**
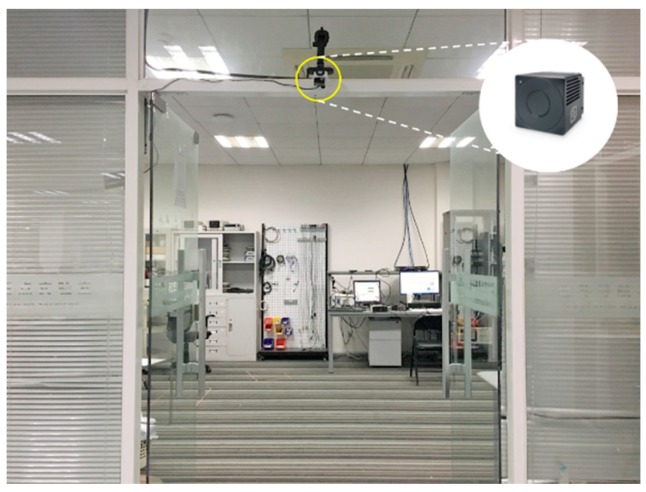
The experimental environment and installation of TOF camera in the laboratory scene.

The TOF camera is equipped at the gate, which is the entrance to an area, at a height of 2.3 m. Figure 8 exhibits the experimental environment and placement of the TOF camera. The simple installation requirements make it suitable for a variety of scenarios. In this paper, we use a SmartToF camera model TC-E2, which acquires depth images with a resolution of 320×240 at a frame rate up to 60 fps. Besides, the TOF camera provides a field of view of 65°×38° in a standard lens. The maximum measurement range is six meters. The mechanical size of the TOF camera is 45 mm×45 mm×39 mm. 

The dataset (http://bat.sjtu.edu.cn/3d-tof-top-view-human-detection-dataset/) comprises of depth image sequences with various numbers of people that flow within the detection area in the same or opposite direction. Each sequence records the total number of people entering and leaving the scene. For 1500 depth images in the dataset, the head positions are manually labeled. Our dataset is collected in common indoor scenes such as laboratories, offices, etc. The proposed dataset allows to evaluate the efficiency and accuracy of our human detection and flow estimation approach in different crowding conditions. We distinguish different scenarios based on flow density. In the simplest scenario, one person passes through the detection area. For complex cases, we imitate crowded people scenes, such as getting on and off the subway during rush hour. In this scene, people stand close to each other and irregularly move in the same or opposite direction. In addition, we collect special scenes such as people waving hands, wearing backpacks and hats to assess the performance of the proposed method for abnormal situations. 

### 4.2. Evaluation on Human Detection

We comprehensively evaluate our method on the proposed dataset and TVHeads (Top-View Heads) [25] dataset, respectively. Our dataset labels the center of head position in each depth image. Besides, TVHeads dataset uses mask images (8 bit), where the heads silhouette is highlighted by improving image contrast and brightness to locate the heads of people who are present below the camera. In this work, we regard the head center point as the ground truth on the TVHeads dataset. The Euclidean distance between head position detection result and annotation point is taken as the evaluation indicator. Three existing outstanding methods are used as the baseline methods for comparisons, including the water filling based method [14], local maxima search method [19], and a semantic segmentation convolutional network for head segmentation U-Net3 [25]. 

Table 1 shows the human detection results using various methods on our proposed dataset, in terms of recall, ${\mathrm{MND}}_{1}$, and NR. Besides, Table 2 exhibits the experimental results on the TVHeads dataset. Recall refers to the proportion of the predicted head position points to the true head position points. The high recall rate ensures that our approach does not miss real proposal points. However, the recall rate is a relatively rough and qualitative evaluation method. Therefore, we propose Mean Nearest Distances (MND) to quantitatively evaluate detection performance, based on the distance between ground truth points and predicted points. It is possible that the numbers of true head points and predicted head points are different. Hence, we match the closest ground truth point and prediction point into a pair by one-to-one mapping based on Euclidean distance, which has the same idea of track assignment filter introduced in Section 3. Similar to Intersection over Union (IoU), the Euclidean distance between the two points is taken as the nearest distance. The L1 norm of MND is used to measure the nearest matching point, denoted as ${\mathrm{MND}}_{1}$. If the accuracy of the human detection method is high enough, the prediction point is close to the ground truth point, which means that the value of ${\mathrm{MND}}_{1}$ is expected to be small. Equation (11) introduces the calculation of ${\mathrm{MND}}_{1}$, and we take the mean value of the L1 norm of MND, for any prediction point $\left(x_{p}^{i},y_{p}^{i}\right)$ and ground truth point $\left(x_{gt}^{j},y_{gt}^{j}\right)$. The ratio of the minimum value and the sub-minimum value of distance from the predicted point to the ground truth point is defined as another indicator, named Nearest Ratio (NR). NR is expected to be small enough to distinguish different prediction points and accurately predict the location of head points.

(11) $${\mathrm{MND}}_{1}=\frac{1}{n}\overset{n}{\underset{j=1}{\sum }}\underset{i}{\min}\;d\left(\left(x_{p}^{i},y_{p}^{i}\right),\left(x_{gt}^{j},y_{gt}^{j}\right)\right)$$

where *n* is the number of matching pairs of true points and prediction points. In other words, *n* is the number of points in which the quantity of head position points is smaller among the two point groups. 

U-Net3 [25] achieved state-of-the-art performance in the semantic head segmentation task based on CNN. Table 3 shows the comparison of human detection result in our method and U-Net3 on the TVHeads dataset, according to the evaluation criteria in U-Net3. Since the goal of U-Net3 is semantic head segmentation task and we focus on human detection, the definition criterion of ground truth is different. Therefore, some locations of our human detection results without entire standard head shapes are not labelled in the TVHeads dataset. In this case, our method performs higher accuracy and recall rate on the TVHeads dataset. Meanwhile, we also have a lower precision and f1-score. 

**Figure 9:**
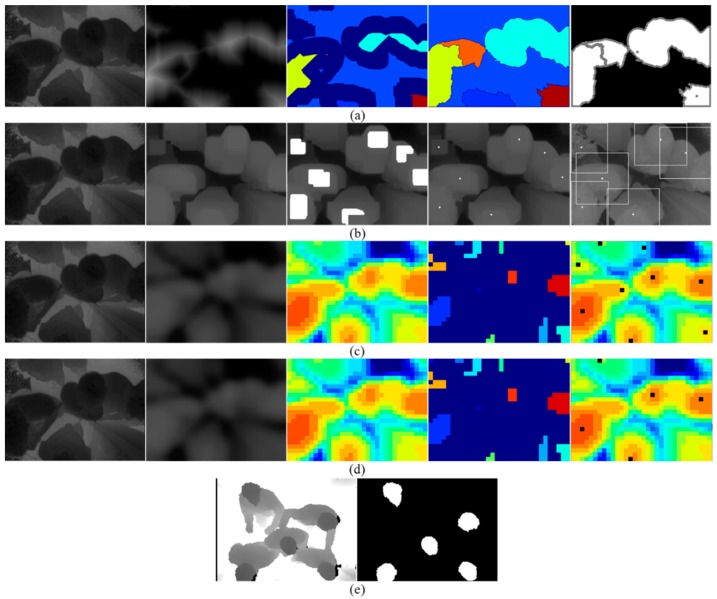
The results of different human detection methods for the same input depth image in our dataset. The input is a typical multiple people scene with occlusion, where five persons in total move in different directions. (**a**) Visualization of processing and results combined with distance transformation and watershed algorithm; (**b**) head central location points clustering based on local maxima search; (**c**) the proposed human detection method based on local pooling and searching; (**d**) the proposed approach combined with classification refinement removing false positive candidates; (**e**) head segmentation results based on convolutional network U-Net3.

In this paper, human detection is a part of the overall system, and the ultimate goal is the accuracy of the flow counting. Therefore, we balance detection performance and computational efficiency, such as shallow CNN for classification refinement, and provide sufficient people location candidates for the subsequent stage. Methods in the tracking module, such as track assignment filter, will compensate for performance issues. In summary, considering the trade-off of detection performance and computational efficiency, our human detection method has the most outstanding performance compared with the existing methods. Figure 9 shows the results of different human detection methods for the same input depth image.

Table 4 describes the head diameter fitting results with different degree of polynomial. We use Root Mean Square Error (RMSE), R-Square ($R^{2}$), R-Square based on RMSE, and classification score to evaluate the performance of fitting. The definition of the indicators is formulated as follows. For the ground truth vector Y and prediction vector $\hat{Y}$:

(12) $$\mathrm{RMSE}\left(Y,\hat{Y}\right)=\sqrt{\frac{1}{n}\overset{n}{\underset{i=1}{\sum }}{\left(y_{i}-\hat{y_{i}}\right)}^{2}}$$

(13) $$R^{2}\left(Y,\hat{Y}\right)=1-\frac{\sum_{i}{\left(\hat{y_{i}}-y_{i}\right)}^{2}}{\sum_{i}{\left(\hat{y_{i}}-\bar{y_{i}}\right)}^{2}}$$

(14) $$R_{rmse}^{}\left(Y,\hat{Y}\right)=1-\frac{RMSE\left(Y,\hat{Y}\right)}{RMSE\left(Y,\bar{y}\right)}$$

where $y_{i}$ is the ground truth diameter of head. $\hat{y_{i}}$ is the prediction result of diameter polynomial fitting. $\bar{y_{i}}$ is the mean value of a set of diameter vector data.

Table 5 traverses the typical size parameters of local searching filters the dataset presents in this paper. According to the experimental results, we selected a 5×5 filter for local searching.

In addition to the indicators for measuring the accuracy of human detection, computational efficiency and real-time performance are also vital in evaluating the performance. Table 6 lists the running time and hardware platform among various approaches.

### 4.3. Evaluation on Flow Estimation

The existing flow estimation method is implemented based on weighted KNN [23] in common, in order to track the location and count the number of people walking along different directions across frames. In this module, we evaluate the performance in various flow estimation methods based on our proposed dataset and TVHeads dataset, respectively. The accuracy of the counting number of people entering and leaving the detection scene is used as an indicator to evaluate the performance of the flow estimation method. 

In addition, the experiment scenarios are divided into the following situations, based on flow density.

- Isolated person walking
- Multiple people walking along different directions, including occlusion situations
- People walking with objects and actions, such as waving hands, holding envelops, wearing caps, etc.

The experiment based on our dataset is carried out in the typical indoor scene of a laboratory and corporate office. In this work, all the experimental data is collected by the users’ daily behavior, such as walking fast, waving hand, casual movements, etc. These scenes can assess challenges faced by the tracking strategy such as slight deformation, rotation, drastic appearance, etc. The comparisons of different strategies are listed in Table 7. According to the experimental results, it is obvious that the proposed combination of track assignment filter and people counting strategy outperforms the other methods.

**Figure 10:**
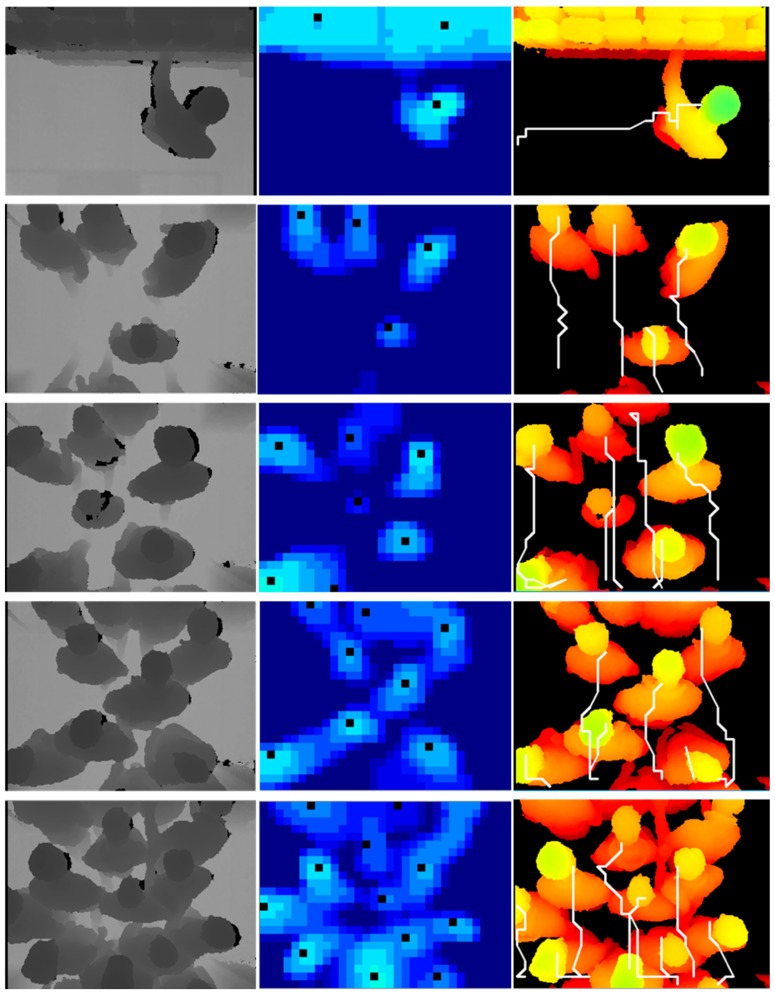
Performance of our human detection and flow estimation approach on the TVHeads dataset with 96.96% AP and 40 fps.

**Figure 11:**
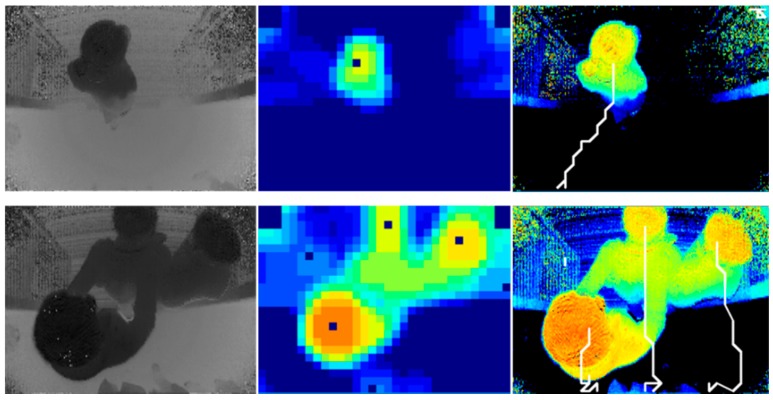
Performance of our human detection and flow estimation approach on our dataset with 97.73% AP and 40 fps.

The depth images in the TVHeads dataset are captured by Kinect, a kind of TOF camera. Due to the large installation height, it has a larger field of view. In other words, the TVHeads dataset provides images that accommodate more people in the scene. In our proposed dataset, the TOF camera is mounted on the door with a lower installation height and a smaller field of view. Therefore, the TVHeads dataset is just complementary to our dataset implementation. It is used to evaluate the performance of our approach for large field-of-view scenes. Figure 10 shows the experimental scenario from single person to multiple people and from simple scene to complex scenes on the TVHeads dataset. Examples of our dataset are shown in Figure 11. Black points in the middle column represent the human positions. Besides, white trajectory lines in the right column show the tracking results by flow estimation module. The experimental results show that in the single person walking scenes, or multi-people walking crowded together with physical contact and occlusion scenes, our proposed algorithm demonstrates robust and outstanding performance in human detection and flow estimation. Thus, the proposed approach outperforms other existing methods with the highest accuracy and fastest running time validated in different datasets.

In the experiment, we notice that different values of threshold ε in track assignment filter have a great impact on the tracking results. In order to get the best results, we select some candidate thresholds and pass over them to select the best one. The corresponding experiment result is recorded in Table 8. In addition, our algorithm not only shows outstanding performance, but also has low computational cost. The average running time of each module in the proposed approach is shown in Table 9. On a CPU platform, it takes 23.10 milliseconds in total to process a depth image frame. This means that our method has very high computational efficiency and real-time performance.

## 5. Conclusions

In this paper, we present a fast and accurate real-time human detection and flow estimation method using depth images captured by a top-view TOF camera. Firstly, a local max-pooling filter resizes the depth image. The human position points are then detected based on local searching and connected component labelling. Besides, classification refinement eliminates the non-head candidates using human morphological features. Finally, a fast track assignment filter based on the punish term composed of height and Euclidean distance features implements the tracking and counting function of flow estimation. Our approach overcomes the over-segmentation and loss-detection in conventional methods, generating head position points with high recall. The proposed track assignment filter and counting strategy cope with the crowded multi-people and occlusion scenes. In addition, a new indoor flow estimation dataset with detailed human location annotations is established. The experiment is carried out in two different datasets. The results demonstrate that the proposed approach outperforms other existing methods with the highest accuracy and fastest running time validated in both datasets. The algorithm runs 23.10 ms per frame on a CPU platform, which means our method has very high computational efficiency and real-time performance.

## Figures and Tables

**Table 1:** Performance of various human detection methods on our proposed dataset.

Method	Recall	${\mathrm{MND}}_{1}$	NR
Water Filling [14]	0.7712	4.3659	0.3373
Local maxima [19]	0.7865	3.6541	0.4179
U-Net3 [25]	0.6749	3.7631	0.4630
Ours	0.9965	1.6813	0.1677

**Table 2:** Performance of various human detection methods on the TVHeads dataset.

Method	Recall	${\mathrm{MND}}_{1}$	NR
Water Filling [14]	0.6220	5.2594	0.6576
Local maxima [19]	0.5324	3.4563	0.5207
U-Net3 [25]	0.9894	1.6499	0.4840
Ours	1	1.7067	0.4562

**Table 3:** Comparison of human detection result in our method and U-Net3 on the TVHeads dataset, according to the evaluation criteria in U-Net3.

Method	Accuracy	Precision	Recall	F1-Score
U-Net3 [25]	0.9945	0.9893	0.9894	0.9893
Ours	0.9953	0.9055	1	0.9504

**Table 4:** Head diameter fitting with different degree of polynomials.

Degree	RMSE	$R^{2}$	$R_{rmse}^{}$	Score
1	11.32	0.86	0.63	0.86
2	6.57	0.94	0.79	0.94
3	6.06	0.96	0.84	0.96
4	6.11	0.95	0.82	0.95

**Table 5:** Human detection results with diverse size of local searching filter.

Filter Size (n)	1	2	5	7	10
Filter Size	3×3	5×5	11×11	15×15	21×21
Recall	0.9965	0.9965	0.9896	0.9721	0.9162
${\mathrm{MND}}_{1}$	1.6822	1.6822	1.7524	1.9500	2.6132
NR	0.1968	0.1758	0.1542	0.1502	0.1776

**Table 6:** Running time and hardware platform in various human detection methods.

Method	Running Time (s)	Platform
U-Net3 [25]	0.6142	NVIDIA GTX 1080 GPU, 8G
Water filling [14]	0.2982	Intel Core i5 CPU, 8G
Local Maxima [19]	0.0758	Intel Core i5 CPU, 8G
Ours	0.0224	Intel Core i5 CPU, 8G

**Table 7:** Performance comparison of different flow estimation methods on the TVHeads dataset and our proposed dataset, respectively.

Dataset	Method	Scene	Accuracy
Ours	KNN [23]	Single person	0.9217
		Multiple people	0.8669
	Ours	Single person	0.9848
		Multiple people	0.9773
TVHeads	KNN [23]	Single person	0.9430
		Multiple people	0.9090
	Ours	Single person	1
		Multiple people	0.9696

**Table 8:** The flow estimation performance with different punish parameter ε.

ε	0	1	2	3
Accuracy	0.5211	0.9028	0.9773	0.9185

**Table 9:** The average running time result of each module of the proposed approach.

Module	Running Time (ms)
Preprocessing	8.18
Local Pooling and Searching	3.48
Classification Refinement	9.97
Flow Estimation	0.66
Visualization	0.81
Total Time	23.10
